# A framework for chiropractic training in clinical preventive services

**DOI:** 10.1186/2045-709X-21-28

**Published:** 2013-08-20

**Authors:** Cheryl Hawk, Marion Willard Evans

**Affiliations:** 1 Logan College of Chiropractic, 1851 Schoettler Rd, 63017 Chesterfield, MO, USA; 2 University of Western States, 2900 NE 132nd Ave, 97230 Portland, OR, USA

**Keywords:** Chiropractic, Preventive health services, Health promotion

## Abstract

The 2010 Patient Protection and Affordable Care Act provides incentives for both patients and providers to engage in evidence-based clinical preventive services recommended by the United States Preventive Services Task Force (USPSTF). Depending upon the application of the new health care act, Doctors of Chiropractic (DC) may be considered to be covered providers of many of these services. It is therefore essential that DCs’ training prepare them to competently deliver them. The aim of this commentary is to describe a framework for training in clinical preventive services, based largely on the USPSTF recommendations, which could be readily integrated into existing DC educational programs.

## Background

### The necessity for preventive care

The United States, despite spending more money on health care than any other country, has a population with shorter life expectancy and greater morbidity than any other wealthy nation [1]. The areas in which the U.S. lags behind its 16 peer nations are all lifestyle-related to a great degree [1]. Clearly this situation requires an approach that engages people in modifying their health behavior, as early as possible, rather than relying on heroic measures once conditions have become chronic and life-threatening. That approach is systematically addressed in the science of health promotion and disease prevention, often simply called “prevention” or “preventive care [2]”.

With the passage of the Patient Protection and Affordable Care Act (PPACA) in 2010, the U.S. government conveyed a strong message that preventive care is essential to reverse the downward spiral of our nation’s health. It also addresses the out-of-control upward spiral of medical expenditures, now more than $2.6 trillion annually [3]. The PPACA takes a number of concrete actions with respect to prevention. Among other actions, it legislates economic incentives for both patients and providers to engage in evidence-based clinical preventive services [4]. Which services are covered is determined by recommendations of the U.S. Preventive Services Task Force (USPSTF) [5].

### Clinical preventive services

Clinical preventive services are services delivered by health care providers to prevent or detect a condition at an early stage. The USPSTF, under the Agency for Healthcare Research and Quality (AHRQ), is mandated by Public Law Section 915 to conduct evidence reviews and make recommendations on clinical preventive services [6]. The USPSTF recommendations therefore represent the “gold standard” for clinical preventive services. Most relevant to this discussion is the fact that all services that the USPSTF rates as A or B—meaning there is certainty of substantial net benefit to patients—are covered under the PPACA. Table 1 summarizes the services rated by USPSTF as A or B. Training is being conducted among primary care practitioners to be sure they are well-versed in these services, and much of this is provided by resources available from the USPSTF [7].

**Table 1:** USPSTF screening and counseling recommendations rated A or B*

**Screening**	**Within scope in most states?**^**1**^	** Relevant course(s)**
Abdominal aortic aneurysm (ultrasound)	no	physical diagnosis
Alcohol misuse	yes	public health/psychology
Breast cancer (mammography)	no	radiology/obstetrics/gynecology
Cervical cancer	no	obstetrics/gynecology
Colorectal cancer	yes	physical diagnosis
Depression^2^	yes	psychology
Diabetes (Type 2)	yes	physical diagnosis
Hearing loss	yes	physical diagnosis/pediatrics
Hypertension	yes	physical diagnosis
Iron deficiency anemia	yes	nutrition
Obesity	yes	nutrition
Osteoporosis	yes	nutrition/geriatrics
Tobacco use	yes	pathology/public health
Lipid disorders	yes	nutrition
Vision	yes	physical diagnosis
**Counseling/interventions**		
Alcohol misuse	yes	public health/psychology
Aspirin for CVD prevention^3^	no	physiology/clinical decision-making
Breastfeeding	yes	obstetrics/gynecology/pediatrics
Folic acid supplement	yes	nutrition
Iron deficiency anemia supplement	yes	nutrition
Obesity weight management^4^	yes	nutrition/psychology
Tobacco use intervention	yes	public health/psychology

* Source: USPSTF, *Guide to Clinical Preventive Services*[2]. Recommendations on screening and counseling for infectious diseases are not included.

^1^ If “no,” training should be included in order to enable appropriate referrals.

^2^ Only if there are systems in place for diagnosis, treatment and follow-up.

^3^ Only if benefits outweigh harm.

^4^ Intensive behavioral intervention to facilitate sustained weight loss.

### Chiropractic and clinical preventive services

The Council on Chiropractic Education states that “As a gatekeeper for direct access to the health delivery system, the doctor of chiropractic’s responsibilities as a primary care physician include wellness promotion, health assessment, diagnosis and the chiropractic management of the patient’s health care needs ([8]^,^p.15).” In spite of this, these concepts are not prominent in the academic standards of chiropractic training institutions. These standards have tended to emphasize conventional concepts of disease diagnosis and treatment, as exemplified in Table 2. However, in 2007 the Council on Chiropractic Education (CCE) added a set of general competencies in wellness to the requirements for chiropractic training [8,9]. In 2012, “best practices” recommendations on chiropractic care provided for the purposes of health promotion, disease prevention and wellness were published, laying out the essential components somewhat more specifically [10]. These core competencies and best practices will be discussed further in this paper.

**Table 2:** **Mandatory topics for Doctor of Chiropractic Programs** (**DCP**)*

**Topic**	**Topic**
adjustive techniques	neurology
anatomy	nutrition/dietetics
biochemistry	obstetrics
biomechanics	orthopedics;
chiropractic principles and practice	otolaryngology
clinical decision making	pathology
dermatology	pediatrics
diagnosis (physical, clinical and laboratory)	physiology
diagnostic imaging	professional practice ethics
first aid and emergency procedures	psychology
geriatrics	public health
gynecology	research methods and procedures
microbiology	spinal analysis

*Source: Council on Chiropractic Education. *Standards for Doctor of Chiropractic Programs and Requirements for Institutional Status*, p. 18 [8].

The PPACA has a nondiscrimination clause that prohibits denial of payment for a covered service by a class of provider acting within their scope of practice, if payment is made to another class of provider for the same service [4]. Depending upon exactly how this clause is interpreted, it could mean that Doctors of Chiropractic (DC) will be able to be paid to deliver most of the USPSTF-recommended clinical preventive services. Thus it is important for DCs to be able to deliver these services in a manner consistent with USPSTF recommendations.

The purpose of this paper is to describe a framework based on the USPSTF recommendations, through which the CCE competencies and 2012 best practices can be operationalized to build a curriculum for clinical preventive services that could be integrated into both DC programs and post-graduate training.

## Discussion

### Current Standards in Chiropractic

The competencies in wellness from the CCE’s *Standards for Doctor of Chiropractic Programs and Requirements for Institutional Status* are shown in Table 3. These specify that, in terms of knowledge, students should know the components of health promotion and the role of the doctor of chiropractic in health promotion, as well as be able to identify health promotion and wellness resources and minimum screening activities for health promotion. With respect to behavior, this document specifies that students be able to perform recommended screening activities and provide patient counseling on health promotion. This document also specifies the topics which must be covered in DC programs.

**Table 3:** Wellness competencies for doctor of Chiropractic Programs*

**Attitudes**	
•	appreciate role of lifestyle, behavior, psychological factors in health and wellness
•	appreciate multidimensionality of wellness
•	appreciate and accept active patient participation
•	explain and emphasize benefits of health promotion on response to treatment
•	appreciate community health and DCs’ role in community health
•	recognize and appreciate impact of environment on patient’s well being
•	appreciate social determinants of health.
**Knowledge**	
•	discuss basic principles and perspectives of health promotion and wellness
•	describe concepts of health promotion in the context of chiropractic health care;
•	describe components of health promotion for the needs of the patient and the public
•	describe the role of the doctor of chiropractic in health promotion
•	relate necessity of lifestyle changes to promote patients’ and the public’s health
•	identify health promotion and wellness resources for patients and the public
•	identify the minimum screening activities for health promotion
•	describe factors related to the leading health indicators (physical activity, overweight and obesity, tobacco, substance abuse, sexual behavior, mental health, injury and violence, environmental quality, immunization, and access to health care)
•	describe issues related to years of healthy life and health disparities.
**Behavior**	
•	communicate effectively with patients about dimensions of health (biological, psychological, social, and spiritual) as part of history taking
•	use appropriate techniques to encourage patient participation in his/her health
•	implement recommended preventive screening activities
•	perform screening and wellness assessments in different age groups
•	provide patient counseling for health promotion and assess its outcomes.

*Source: Council on Chiropractic Education. *Standards for Doctor of Chiropractic Programs and Requirements for Institutional Status*, p.46-48 [8].

Furthermore, in 2013, the CCE refined the wellness competencies in their new meta-competency approach [11]. The new Meta-competency 3 is “health promotion and disease prevention”. Table 4 summarizes the required components and outcomes for this meta-competency. Colleges must demonstrate to CCE where in their curriculum they achieve these outcomes. This meta-competency is basically a directive for chiropractic colleges to train students in screening for risk factors and counseling on health behavior. As shown in Table 2, there are no topics explicitly addressing the “wellness” competencies. However, it is conceivable that the necessary information could be combined with topics such as chiropractic principles and practice, public health and nutrition, for counseling, and diagnosis for screening. The Standards do not require a specific course for any topic; the institution needs to demonstrate that all the topics are included in coursework. Thus there is considerable flexibility in how preventive services training can be integrated into existing courses.

**Table 4:** **Meta**-**competency 3**: health promotion and disease prevention*

	
**Required components**	Identify areas for health improvement (that is, screen for disease and risk factors)
	Address appropriate hygiene (that is, advise on health behavior)
	Coordinate strategies with other providers.
	Identify public health issues relevant to patients
**Outcomes**	Document management of health risks.
	Explain health risk factors to patients.
	Provide recommendations on patients’ health status, behavior and lifestyle.
	Recommend/provide resources and instruction to assist health behavior change.
	Recommend dietary approaches to restore, maintain or improve patient’s health.
	Implement appropriate hygiene practices in the clinical environment.
	Communicate strategies to other treating providers.

*Source: Council on Chiropractic Education. Accreditation Standards, Principles, Processes & Requirements for Accreditation. Scottsdale, AZ; 2013.

The National Board of Chiropractic Examiners (NBCE) covers the following topics on board examinations DCs must pass in order to be licensed:

Community Health and Wellness (11% of NBCE board examination, Part II score)

•Public health organizations

•Healthy People initiatives

•Screening activities for health promotion

•Tobacco, alcohol and substance abuse

•Exercise and healthy diet for obesity

•Behavior theories and lifestyle change

•Wellness counseling

The “best practices” recommendations, based on the results of a formal consensus process conducted through a multidisciplinary Delphi panel, are summarized in Table 5[10]. Obviously these recommendations are consistent with, although more detailed than, both the general CCE competencies and the more specific topics listed by the NBCE. The “best practice” recommendations operationalize some of the broader items in the CCE and NBCE documents. For example, the “best practice” recommendations state that all patients should be screened for obesity/overweight using the Body Mass Index (BMI), and that tobacco cessation advice should at least include provision of a quit line phone number [10]. This document also states that DCs should be familiar with nationally recognized recommendations on disease screening and health promotion counseling, such as those made by the USPSTF [10].

**Table 5:** **Summary of** “**best practice**” recommendations for chiropractic wellness care*

**General**	
Be familiar with nationally recognized recommendations on disease screening and health promotion counseling, such as those made by the USPSTF.	
**Screening for risk factors**—**all patients**	
•	Obesity/overweight, as assessed by Body Mass Index (BMI)
•	Physical inactivity or sedentary behaviors
•	Tobacco use
•	Hypertension
**Other considerations**	
•	Awareness of symptoms of depression and availability of appropriate referral system for depression or other psychological issues.
•	Awareness of signs and symptoms of skin cancer and availability of appropriate referral system.
**Health promotion information**, **advice or referral to all patients for whom screening indicates that they are at risk:**	
•	Physical activity appropriate for the individual
•	Tobacco cessation, at least through provision of quit line phone number.
•	Healthy diet
•	Weight management appropriate for the individual.
**Other considerations**	
•	Although immunization is not within the chiropractic scope of practice, if patients ask for information, they should be referred to or receive balanced, evidence-based information from credible resources such as the Centers for Disease Control.

*Source: Hawk C, Schneider M, Evans MW, Jr., Redwood D. Consensus process to develop a best-practice document on the role of chiropractic care in health promotion, disease prevention, and wellness. *J Manipulative Physiol Ther*. Sep 2012;35(7):556–567 [10].

### USPSTF recommendations as a framework

A pragmatic approach to integrating clinical preventive services would be to use the USPSTF recommendations as the basis for operationalizing the CCE standards and “best practices”. Table 1 lists the USPSTF recommendations rated A or B (excluding infectious diseases), [2] along with whether or not they are generally within chiropractic scope of practice, and courses within which they might most readily be integrated. It should be noted that the USPSTF recommendations encompass all the current chiropractic standards—CCE competencies, “best practice” recommendations and NBCE topics.

### Clinical experience in counseling/advising patients

Students within a doctor of chiropractic program already have opportunities to counsel patients on behavior change during their internship. For example, since the majority of U.S. adults are overweight or obese and get less than the recommended amount of physical activity, [2] it is likely that students will encounter such patients. We suggest that colleges might use the USPSTF recommendations as a framework to ensure that opportunities to counsel patients on the recommended topics be included, through use of standardized patients if necessary [12]. This would simultaneously address the CCE behavioral competencies, NBCE topics and “best practices”. The standardized patient encounter could be as simple as having a patient present with chronic spinal pain and a history of cigarette smoking for several years, or the need to implement age-appropriate physical activity guidelines. In addition, at the USPSTF website there is also information on whom to contact to access information about how to test students on preventive services using a Preventive Care Objective Structured Clinical Examination (OSCE). This OSCE was developed jointly by the Indiana University School of Medicine and the Indiana Area Health Education Center (AHEC). Current use of OSCEs is common in chiropractic colleges; our suggestion is merely to extend their use to include prevention counseling.

This approach would provide assurance that DCs are trained to provide the preventive services within their scope of practice that are well-documented to have an important net benefit for patients’ health. Furthermore, it would provide documentation that DCs are capable of providing the services covered by the PPACA, which would therefore help in making a case for DCs to be covered providers of these services.

### Challenges to implementing the wellness competencies

In a curriculum which is already overfull with didactic coursework, many administrators and faculty may feel that it is impossible to add additional material. However, the CCE requirement to do exactly that may induce some creative thinking. Reducing some basic science material, such as microbiology or biochemistry, could make room for more material on health behavior and health education. In terms of clinical experience, it seems quite feasible to simply “get more mileage” out of existing standardized and actual patients. That is, rather than restricting the history and examination to vital signs, orthopedic, neurological, postural, or palpatory findings, simply broaden them to include BMI assessment, health habits and other lifestyle factors. In fact, many and perhaps most chiropractic colleges already do include lifestyle in the history. The next step, not uniformly taken as yet, would be to require students to counsel patients on changing risky health behaviors. These patients may be presenting for musculoskeletal complaints or, like many patients in chiropractic teaching clinics, may be presenting for “wellness care”. These are opportunities to screen for health risks, particularly those which are extremely common, such as physical inactivity and overweight/obesity, and to counsel patients when the risks are present. Thus, current patients (both standardized and actual) could provide opportunities for screening and counseling at the same time that they are being treated for symptoms/conditions.

### Resources for faculty

In the end, success in integrating training in clinical preventive services into chiropractic education depends upon the faculty. Since many chiropractic college faculty do not have specific training in clinical preventive services, it is essential that colleges develop faculty in-services or support selected faculty in pursuing appropriate coursework, in areas such as public health, health education, health psychology, or health/wellness coaching. The USPSTF’s Pocket Guide to Clinical Preventive Services, along with other electronic resources, is available at http://www.USPreventiveServicesTaskForce.org.

Because of its pivotal role in the implementation of the PPACA, the USPSTF provides a wealth of teaching resources available to health professions training institutions and/or other organizations [7]. They were created to assist in the training of primary care providers in use of the recommended preventive services in practice. The American Association of the Colleges of Osteopathic Medicine’s Task Force on Integrating Preventive Medicine into Medical and Health Professions Curricula created one- and two-hour PowerPoint presentations for health care professionals titled “Putting Prevention into Practice”. These are case-based tutorials which serve as an introduction to use of the USPSTF recommendations.

Combining the existing USPSTF resources with books and other resources, and integrating modules into appropriate existing courses, as well as with clinical training using standardized patients and OSCEs, would not require extensive curriculum revision. Preparing faculty through a series of in-services, again using readily accessible resources, could also be integrated into existing academic schedules. Using the USPSTF recommendations as a framework for health promotion and disease prevention training could assist in operationalizing the CCE requirements for this competency. Also, since they are in broad use by all health professions, they could assist in interprofessional communications between doctors of chiropractic and other providers.

## Conclusion

Combining the framework of the USPSTF recommendations with the publicly available USPSTF resources should be relatively easily adapted to different chiropractic colleges’ needs and preferences. It would ensure that graduates are prepared to contribute substantively to the national effort toward disease prevention and promoting the health of all Americans.

## Competing interests

The authors have recently published a book on the topic of clinical preventive services and developed a certification program with the National Wellness Institute on health promotion and wellness. The authors declare that they have no competing interests.

## Authors’ contributions

CH conceived of the paper and drafted the manuscript. WE contributed to the concepts and references and to writing the manuscript. Both authors read and approved the final manuscript.
